# Exploring the benefits of in-diet versus repeated oral dosing of saracatinib (AZD0530) in chronic studies: insights into pharmacokinetics and animal welfare

**DOI:** 10.3389/fvets.2023.1297221

**Published:** 2023-11-09

**Authors:** Suraj S. Vasanthi, Nyzil Massey, Suresh N. Nair, Jonathan P. Mochel, Lucas Showman, Thimmasettappa Thippeswamy

**Affiliations:** ^1^Department of Biomedical Sciences, College of Veterinary Medicine, Iowa State University, Ames, IA, United States; ^2^Department of Veterinary Pharmacology and Toxicology, College of Veterinary and Animal Sciences, Kerala Veterinary and Animal Sciences University, Thrissur, India; ^3^Precision One Health, Department of Pathology, College of Veterinary Medicine, University of Georgia, Athens, GA, United States; ^4^W.M. Keck Metabolomics Research Laboratory, Iowa State University, Ames, IA, United States

**Keywords:** saracatinib, Src tyrosine kinases, serum, LC–MS/MS, hippocampus, pharmacokinetics

## Abstract

Saracatinib/AZD0530 (SAR), a Src tyrosine kinase inhibitor, mitigates seizure-induced brain pathology in epilepsy models upon repeated oral dosing. However, repeated dosing is stressful and can be challenging in some seizing animals. To overcome this issue, we have incorporated SAR-in-Diet and compared serum pharmacokinetics (PK) and brain concentrations with conventional repeated oral dosing. Saracatinib in solution or in-diet was stable at room temperature for >4 weeks (97 ± 1.56%). Adult Sprague Dawley rats on SAR-in-Diet consumed ~1.7 g/day less compared to regular diet (16.82 ± 0.6 vs. 18.50 ± 0.5 g/day), but the weight gain/day was unaffected (2.63 ± 0.5 g/day vs. 2.83 ± 0.2 g/day). Importantly, we achieved the anticipated SAR dose range from 2.5–18.7 mg/kg of rat in response to varying concentrations of SAR-in-Diet from 54 to 260 ppm of feed, respectively. There was a strong and significant correlation between SAR-in-Diet dose (mg/kg) and serum saracatinib concentrations (ng/ml). Serum concentrations also did not vary significantly between SAR-in-Diet and repeated oral dosing. The hippocampal saracatinib concentrations derived from SAR-in-Diet treatment were higher than those derived after repeated oral dosing (day 3, 546.8 ± 219.7 ng/g vs. 238.6 ± 143 ng/g; day 7, 300.7 ± 43.4 ng/g vs. 271.1 ± 62.33 ng/g). Saracatinib stability at room temperature and high serum and hippocampal concentrations in animals fed on SAR-in-Diet are useful to titer the saracatinib dose for future animal disease models. Overall, test drugs in the diet is an experimental approach that addresses issues related to handling stress-induced variables in animal experiments.

## 1. Introduction

Saracatinib, also known as AZD0530, is a selective inhibitor of Fyn or Src family of nonreceptor tyrosine developed by AstraZeneca. Fyn/Src is highly expressed in proliferating cells such as cancerous cells (1, 2) neurons and glial cells of the central nervous system in response to brain insults such as exposure to neurotoxins (3) or seizures (4, 5) or in chronic neurodegenerative diseases such as Alzheimer’s disease (6). Considering the role of Fyn/Src kinases in cell proliferation, saracatinib has been in clinical trials for various types of cancers, such as bone, ovarian, and breast cancer (7–9). Excessive production of Fyn/Src and its phosphorylation in reactive glial cells or neurons causes hyperexcitability, neuroinflammation, and neurodegeneration (10, 11). Therefore, saracatinib has been tested as a disease modifier in experimental models of epilepsy and AD (3, 12) and in clinical trials for AD and Parkinson’s disease (13, 14).

Saracatinib is orally active, crosses the blood brain barrier, and is a potent modulator of ABCB1-mediated multidrug resistance (9, 15). However, long-term treatment with this drug at an optimal dose is required for debilitating and chronic diseases such as cancer and neurodegenerative diseases to achieve beneficial effects with minimum adverse effects. In humans, saracatinib has been tested in clinical trials for some of these conditions for varying periods (8, 9). In experimental models of epilepsy, we tested repeated oral dosing of saracatinib for about a week and observed some disease-modifying effects depending on the severity of the disease and the models of epilepsy (3, 16). In the kainate (KA) model of temporal lobe epilepsy, treating for a week with saracatinib was sufficient to mitigate long-term effects such as reactive gliosis, neurodegeneration, epileptiform spiking, and spontaneously recurring seizures (3). However, such protective effects of saracatinib treatment for a week were limited in organophosphate (OP) induced epilepsy models, perhaps due to widespread peripheral effects and extended duration of body clearance of OP (16). Furthermore, acute exposure to OP impacts gut dysbiosis (17), in contrast to the more centrally localized effect of KA, therefore, the drugs administered via oral gavage for a shorter duration may have limited effects in OP models. Chronic and debilitating diseases such as cancer, AD, PD, and epilepsy require long-term treatment, and oral medication is ideal for attaining optimum plasma concentration from a translational perspective.

In experimental models, daily administration of test drugs by oral gavage for long term is tedious, impractical, and stressful for animals due to repeated handling, which could confound the real effects of interventional drugs. To mitigate these issues, we incorporated saracatinib in rat chow to achieve the required dose during the 24 h feeding cycle and compared the feed consumption, weight gain, and the pharmacokinetics of saracatinib in serum and brain with repeated daily oral dosing of saracatinib for a week. We also tested the serum saracatinib concentrations when the animals were fed different concentrations of saracatinib in the diet for long term. LC–MS/MS confirmed the saracatinib concentrations in the formulated diet. The serum saracatinib in both groups showed a strong correlation from day 2 through day 7, and there were no significant differences between groups. Overall, saracatinib stability at room temperature and high serum and hippocampal concentrations in animals fed on SAR-in-Diet are useful for titrating the required dosing regimen in chronic disease models. Incorporating test drugs in the diet is a translational approach and abates stress-induced variables in animal experiments.

## 2. Materials and methods

### 2.1. Animal studies

Twenty eight adult male Sprague Dawley rats (8 weeks old; 250-300 g; Charles River, United States) were used in the study. Animals were housed in individual cages with a 12-h light–dark cycle in an enriched environment. The experiments were conducted after 2–3 days of acclimatization as per the approved protocols by the Institutional Animal Care and Use Committees (IACUC protocol: 21–109) and complied with the NIH ARRIVE Guidelines for the Care and Use of Laboratory Animals. Blood sampling was done by retro-orbital puncture as per IACUC protocol. Maximal blood collection was limited to less than 7.5% of the animal’s body weight in one week. The serum was separated by centrifugation at 1,000–1,500 × g for 10 min and stored at −80°C until further analysis. All animals were euthanized at the end of the study with pentobarbital sodium and phenytoin sodium (100 mg/kg, i.p.) as per the American Veterinary Medical Association Guidelines for euthanasia. Tissue samples were collected and stored at −80°C until further analysis.

**Figure 1:**
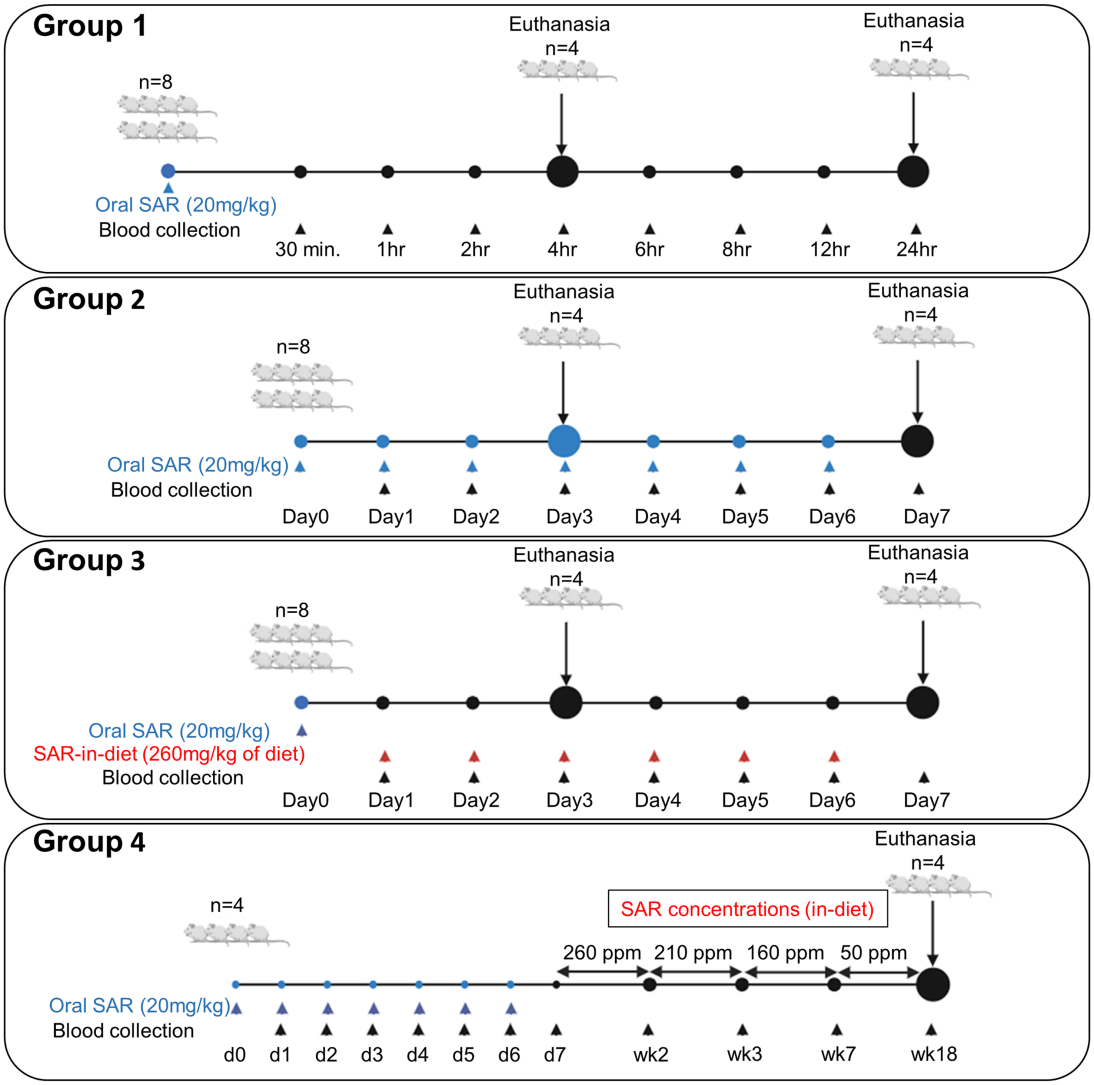
Experimental design for a single dose pharmacokinetics of saracatinib [24 h study, **(A)**], repeated daily oral dose [for 7 days, **(B)**], SAR-in-Diet [for 7 days, **(C)**], and for long term studies **(D)**.

The experimental design is illustrated in Figure 1. SAR-in-Diet at 260, 210, 160, and 50 ppm was prepared by LabDiet (Lan O’Lakes, Inc) to achieve the saracatinib dose range of 20-5 mg/kg of rat. The required concentration of saracatinib to be incorporated in the rat chow was estimated based on the average daily food consumption (18-21 g/day) from eight male adult rats of the same age (8 weeks to start with) and a similar weight range (206–230 g). Saracatinib was prepared in 0.5% hydroxypropyl methylcellulose at 5 mg/mL and added to the diet at 260, 210, 160, and 50 ppm. The Saracatinib incorporated diet and the control (regular rat chow) diet had similar composition/ingredients and was purchased from the same source, Purina 5P07 (Prolab RMH 1000). Saracatinib >99% pure by Liquid Chromatography–Tandem Mass Spectrometry (LC–MS/MS) was supplied by AstraZeneca under the Open Innovation Program.

The animals were randomized into four experimental groups (*n* = 4–8). In group 1 (24 h study; Figure 1A), eight animals received a single oral gavage of saracatinib (20 mg/kg), and the blood sampling was done at 0.5, 1, 2, 4, 6, 8, 12, and 24 h post-treatment. Four animals each were euthanized at 4 h and 24 h, respectively. In group 2 (7-day repeated oral dosing study; Figure 1B), eight animals received repeated daily oral dosing of saracatinib (20 mg/kg of rat) at 24 h intervals; the blood sampling was done daily at 24 h intervals before the next dosing. Four animals were euthanized 24 h after the third repeated oral dosing, i.e., day 3 and the other four animals were euthanized at day 7. Group 3 (7-day SAR-in-Diet study; Figure 1C), received a single oral gavage of saracatinib (20 mg/kg of rat) followed by 24 h later SAR-in-Diet for 7 days (260 ppm). The blood sampling was done at 24 h post oral and daily at 24 h intervals for 7 days while the animals were on SAR-in-Diet. Four animals each were euthanized on day 3 and 7. Group 4 (Figure 1D) included four animals that were given 7 repeated doses of oral saracatinib (20 mg/kg/day for a week) followed by *ad-libitum* SAR-in-Diet at varying concentrations fed for up to 18 weeks (260 ppm diet for 2 weeks, 210 ppm diet for a week, 160 ppm for 4 weeks, and 50 ppm diet for 11 weeks) The tapering concentrations of saracatinib in the diet from 260 to 50 ppm was done to target a daily saracatinib dose rate in the range of 20–5 mg/kg respectively, in a 24 h feeding cycle. Blood samples were collected at the end of each diet change.

### 2.2. Serum and hippocampal samples preparation for LC–MS/MS

Samples were submitted to the W.M. Keck Metabolomics Research Laboratory (Office of Biotechnology, Iowa State University, Ames, IA) for targeted saracatinib LC–MS/MS quantification. Serum and hippocampus preparations were conducted using a modified version of the previously available extraction and sample preparation methods previously established (18, 19). 50–75 μL or 50–75 mg wet weight of each sample was added to microcentrifuge tubes. A Reagent control sample was generated for each sample batch, 75 μL of 0.9% NaCl which was extracted in the same fashion as the samples. The weighed samples were then spiked with the internal standard, 1.25 μg of S-Hexylglutathione (Sigma-Aldrich CO., St. Louis, MO), added as 10 μL of from a 0.125 mg/mL solution in LC–MS grade 9:1 methanol: acetonitrile (Fisher Scientific, Waltham, MA). The extraction was initiated by the addition of 0.4 mL of 90% ice-cold LC–MS grade methanol with 10% LC–MS grade water (Fisher Scientific, Waltham, MA). For the hippocampus samples, after adding the 90% methanol to the samples, two 2.4 mm metal beads were added to each sample, the tissue was then homogenized using a Bead Mill 24 Homogenizer (Thermo Fisher Scientific, Inc., Waltham, MA, United States). All samples were then vortexed 10 s and placed on ice for 10 min before the addition of 1.0 mL of 3:1 of LC–MS grade acetonitrile: water (Fisher Scientific, Waltham, MA). The samples were again vortexed for 10 s before being placed in an ice-cold sonication water bath (Branson Ultrasonics, Brookfield, CT) for 10 min. Samples were vortexed for 10 min and centrifuged for 7 min at maximum speed (16,000 g). The serum sample supernatant extracts were transferred to LC vials and subjected directly to LC–MS/MS analysis. The hippocampus samples sample extracts were filtered with 0.2-micron centrifugal filters (Cat. No. UFC30LG25, Millipore Sigma, Burlington, MA) prior to LC–MS/MS analysis. A saracatinib (AstraZeneca, Cambridge, England, United Kingdom) standard curve with a range of 0.49 to 1,000 ng per sample was prepared as 1:1 serial dilutions in 90% methanol before being extracted and subjected to LC–MS/MS analysis in the same manner as the biological samples.

### 2.3. Food pellet sample preparation for LC–MS/MS

Samples were submitted to the W.M. Keck Metabolomics Research Laboratory (Office of Biotechnology, Iowa State University, Ames, IA) for targeted saracatinib LC–MS/MS quantification. One or two food pellets (1.5–3 g) were homogenized by mortar-and-pestle before being transferred and weighed in glass tubes with Teflon-lined screw caps. A reagent control sample was generated for each sample batch which was prepared in the same fashion as the samples. The weighed samples were then spiked with the internal standard, 250 μg of S-Hexylglutathione (Sigma-Aldrich CO., St. Louis, MO), added as 1.0 mL of from a 0.25 mg/mL solution in LC–MS grade 9:1 methanol: acetonitrile (Fisher Scientific, Waltham, MA). The extraction was initiated by the addition of 8 mL of 90% LC–MS grade methanol with 10% LC–MS grade water (Fisher Scientific, Waltham, MA). The samples were then vortexed 10 s and allowed to sit on ice for 10 min before the addition of 12 mL of 5:1 of LC–MS grade acetonitrile: water (Fisher Scientific, Waltham, MA). The samples were again vortexed for 10 s before being placed in an ice-cold sonication water bath (Branson Ultrasonics, Brookfield, CT) for 10 min. Samples were vortexed for 10 min and centrifuged for 30 min at 900 g). The food pellet sample supernatant extracts were transferred to LC vials and subjected directly to LC–MS/MS analysis. A saracatinib (AstraZeneca, Cambridge, England, UK) standard curve with a range of 75 to 6 00 μg per sample was prepared as 1:1 serial dilutions in 90% methanol before being extracted and subjected to LC–MS/MS analysis in the same manner as the food pellet samples.

### 2.4. LC–MS/MS

LC separations were performed with an Agilent Technologies 1,290 Infinity Binary Pump UHPLC instrument equipped with an Agilent Technologies Eclipse C18 1.8 μm 2.1 mm × 100 mm analytical column that was coupled to an Agilent Technologies 6,540 UHD Accurate-Mass Q-TOF mass spectrometer (Agilent Technologies, Santa Clara, CA). A volume of 6 μL of each sample (0.5 μL of food pellet samples) was injected into the LC system. Chromatography was carried out at 40°C with a flow rate of 0.400 mL/min. Running solvents were A: water with 0.1% formic acid and B: acetonitrile with 0.1% formic acid. Initial solvent conditions were 1% B which increased on a linear gradient to 50% B over 7 min before increasing to 100% B over 2 min, 100% B was held for 6 min before returning to 1% B over 2 min. A 5-min post run at 1% B was conducted after each LC–MS/MS acquisition. Saracatinib was detected using electrospray ionization in positive ionization mode. Nitrogen was used as the service gas for the ion source with a drying gas flow rate of 12 L/min at a temperature of 350°C, a nebulizing pressure of 25 psi, and a sheath gas flow of 11 L/min at 400°C. The capillary and nozzle voltages were 4,000 and 1,750 volts, respectively. The mass spectrometer was operated in high resolution (4Gz) mode with a scan range from m/z 100 to m/z 1,700 for MS and m/z 60 to m/z 350 for MS/MS. An acquisition rate of 10 spectra per second was used for MS and 5 spectra per second for MS/MS, respectively. During LC–MS data acquisition, reference masses were monitored for continuous mass calibration: m/z 121.050873 with m/z 922.009698. Two targeted MS/MS selections were acquired: for saracatinib m/z 542.2 with a narrow isolation width and a collision energy of 25 eV; for the internal standard m/z 392.2 with a narrow isolation width and a collision energy of 25 eV. Data evaluation and peak quantitation were performed using Agilent MassHunter Qualitative Analysis (version 10.0) and Agilent MassHunter Quantitative Analysis (version 10.0) software (Agilent Technologies, Santa Clara, CA). Target peaks were quantified using the LC–MS/MS extracted ion chromatographs for saracatinib 542.2- > 127.1218 ± 50 ppm and the internal standard 392.2- > 246.1143 ± 50 ppm. Saracatinib quantification was finally determined by relative abundance to the internal standard and linear saracatinib standard curve before being made relative to the measured sample mass and volumes. The limit of detection (LOD) and quantification (LLOQ) were determined by the signal-to-noise ratio. The LLOQ was determined by the concentrations of spiked calibration standards.

### 2.5. Pharmacokinetic parameter analysis

A noncompartmental pharmacokinetic (or statistical moments) analysis was performed using PKanalix software ©Lixoft version 2023 to derive the following pharmacokinetic parameters for saracatinib after a single oral dose (20 mg/kg) 24 h study: elimination half-life (T_1/2_), elimination rate constant (K_el_), maximum concentration (C_max_), time to reach maximum concentration (T_max_), apparent volume of distribution (V_d/F_), apparent systemic clearance (CL/F). The first saracatinib concentration post dose below the LLOQ was inferred to be LLOQ/2, and subsequent data points were excluded from the analysis. Area under concentration vs. time curves was determined using the linear/log trapezoidal rule. The slope of the terminal phase for saracatinib (λz) was derived by linear regression between *Y* (log(concentrations)) and the *X* (time) using a 1/*Y*^2^ weighting method.

### 2.6. Experimental rigor and statistics

The experimental groups and samples were coded/blinded until the raw data were compiled and analyses were completed. We used GraphPad Prism 9.0 for statistical analysis. Normality tests were performed using the Shapiro–Wilk test. Based on the normality of the data, t-test or Mann–Whitney test was applied to compare data between the two groups. A mixed-effects model was performed to determine differences at different time points. Further statistical details are included in the figure legends of the corresponding figures.

## 3. Results

The experimental design is illustrated in Figure 1.

### 3.1. Feed consumption and bodyweight comparison between groups

**Figure 2:**
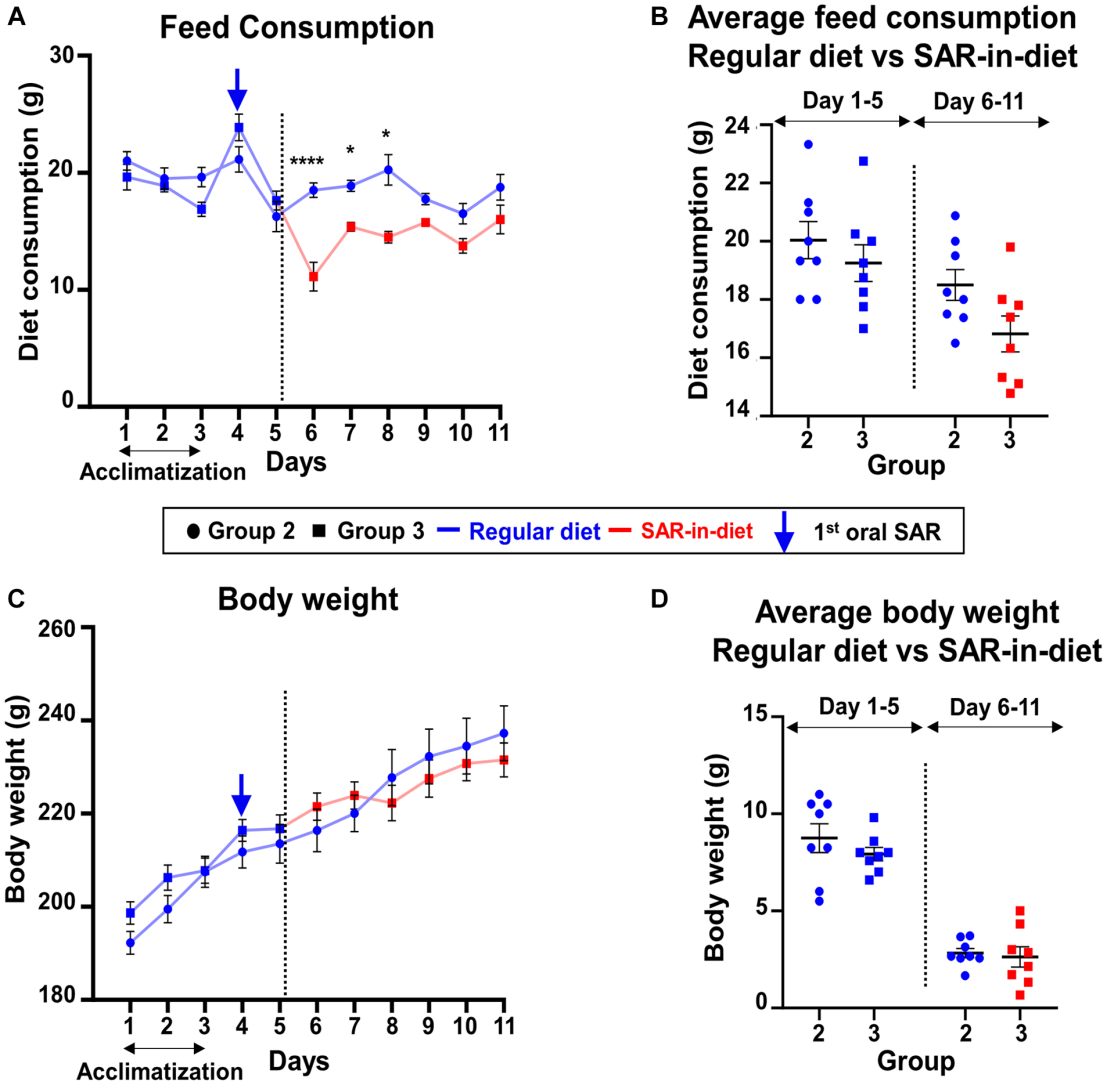
Comparison of feed consumption **(A,B)** and weight gain **(C,D)** between the animals that were on regular diet (Group 2) versus SAR-in-Diet (Group 3) for a week. *n* = 4*–*8 **(A,C)** mixed-effects analysis (Šídák’s multiple comparisons test); *n* = 8 **(B,D)** two-way ANOVA (Tukey’s multiple comparisons test); *p < 0.05, ****p < 0.0001.

In this study, the daily food intake and body weight during the one week experimental period were compared between the animals fed on a regular diet (Group 2) and SAR-in-Diet (Group 3). When the daily food consumption was compared between the two groups, the SAR-in-Diet group consumed significantly less food on days 6–8 (Figure 2A); however, there were no significant differences in the average diet consumption before and after the first saracatinib oral dosing (Figure 2B). Interestingly, despite the reduction in daily food consumption in SAR-in-Diet group, there were no significant differences in the weight gain between the groups at any day (Figures 2C,D).

### 3.2. Authentication of saracatinib and its stability at room temperature in solution or when it was incorporated into the diet: measured by LC–MS/MS

**Figure 3:**
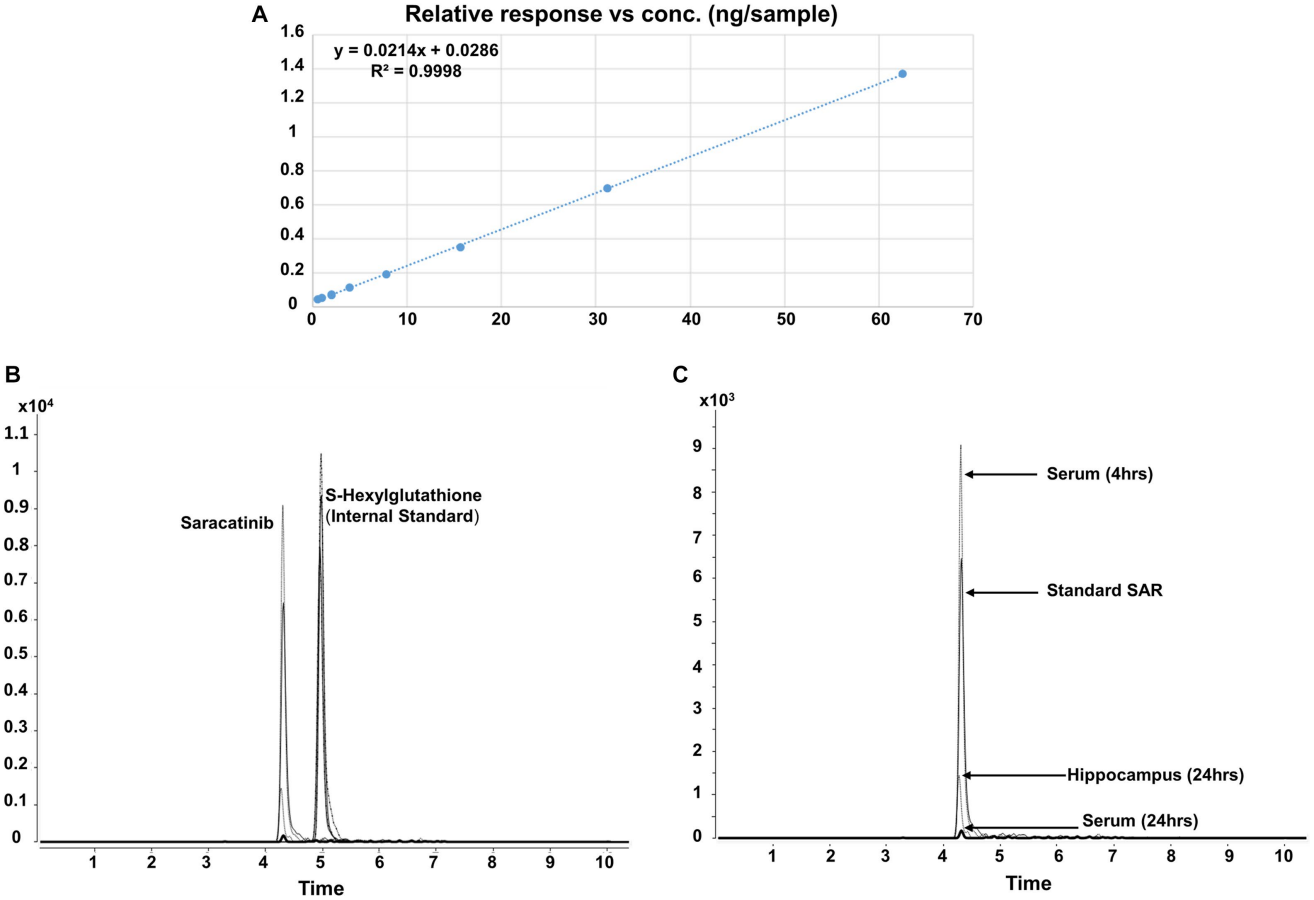
Method validation for saracatinib using LC–MS/MS. Response linearity of saracatinib **(A)**, chromatogram of saracatinib and internal standard (S-Hexylglutathione) **(B)**, chromatogram of saracatinib in the serum, the hippocampus and the standard saracatinib **(C)**.

Before the saracatinib was tested in animals, its identity and purity (>99%) was determined by LC–MS/MS. We first determined response linearity for saracatinib (Figure 3A). The retention time for saracatinib and the internal standard S- Hexylglutathione was 4.315 and 4.992 min, respectively (Figures 3B,C). When peak area (*y*) was plotted against the ascending saracatinib standard, a good correlation coefficient *R*^2^ of 0.9998 was obtained, which was within the accepted range and showed a linear relationship. The slope (m) and intercept (c) of the calibration curve were 0.0214 and 0.0286, respectively. The LOD and LLOQ of the method was 7.35 ng/mL or ng/g and 22.65 ng/mL or ng/g, respectively. The SAR-in-Diet or in solution at room temperature was stable for >4 weeks (97 ± 1.56%) as detected by LC–MS/MS.

### 3.3. Serum pharmacokinetics and hippocampal concentrations of saracatinib after a single oral dose (20 mg/kg) 24 h study

**Figure 4:**
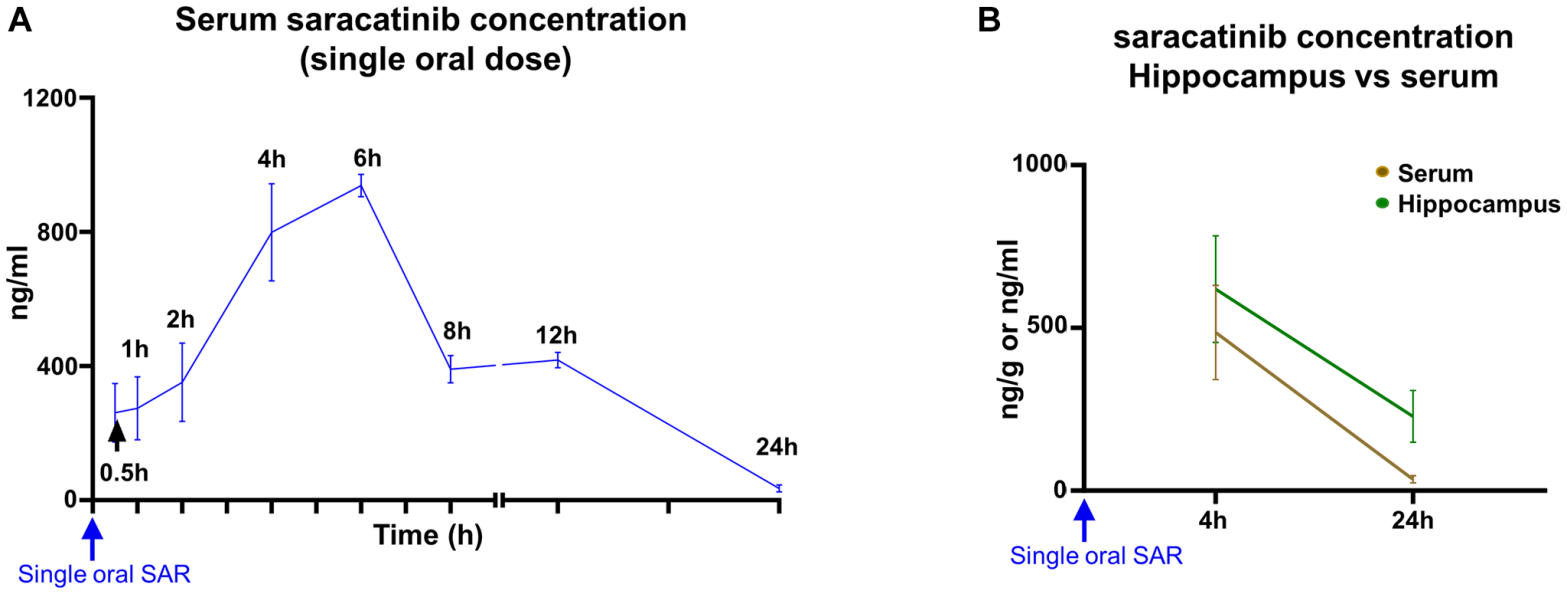
Mean (± SEM) serum and hippocampal concentrations of saracatinib after a single oral dose (20 mg/kg). *n* = 4–8 **(A)**; *n* = 4 **(B)** mixed-effects analysis (Šídák’s multiple comparisons test).

After a single bolus dose of saracatinib (20 mg/kg, oral gavage), the absorption of the drug seems biphasic. The initial absorption from the GI lumen was slow for up to 2 h, followed by a steep increase in the rate of absorption. The peak serum concentration attained by saracatinib was 954.42 ng/mL at 6 h (Figure 4A). The mean volume of distribution was 15.59 L/kg, and the clearance was 2.83 L/h/kg. Saracatinib was detected in the serum up to 24 h after a single oral dose. The estimated elimination half-life of saracatinib was 4.12 h with a rate constant of 0.18 per hour. The area under the concentration-time curve (AUC) from 0 to 24 h was 6992.15 (ng/mL*h) (Supplementary Table S1). The hippocampal levels were > 500 ng/g at 4 h, and by 24 h, the concentrations dropped to nearly half compared to 4 h. The hippocampal concentrations were higher than the serum concentrations at 4 h and 24 h (Figure 4B).

### 3.4. Serum and hippocampal concentrations of saracatinib in repeated daily dosing and SAR-in-Diet groups in the 7-day study groups

**Figure 5:**
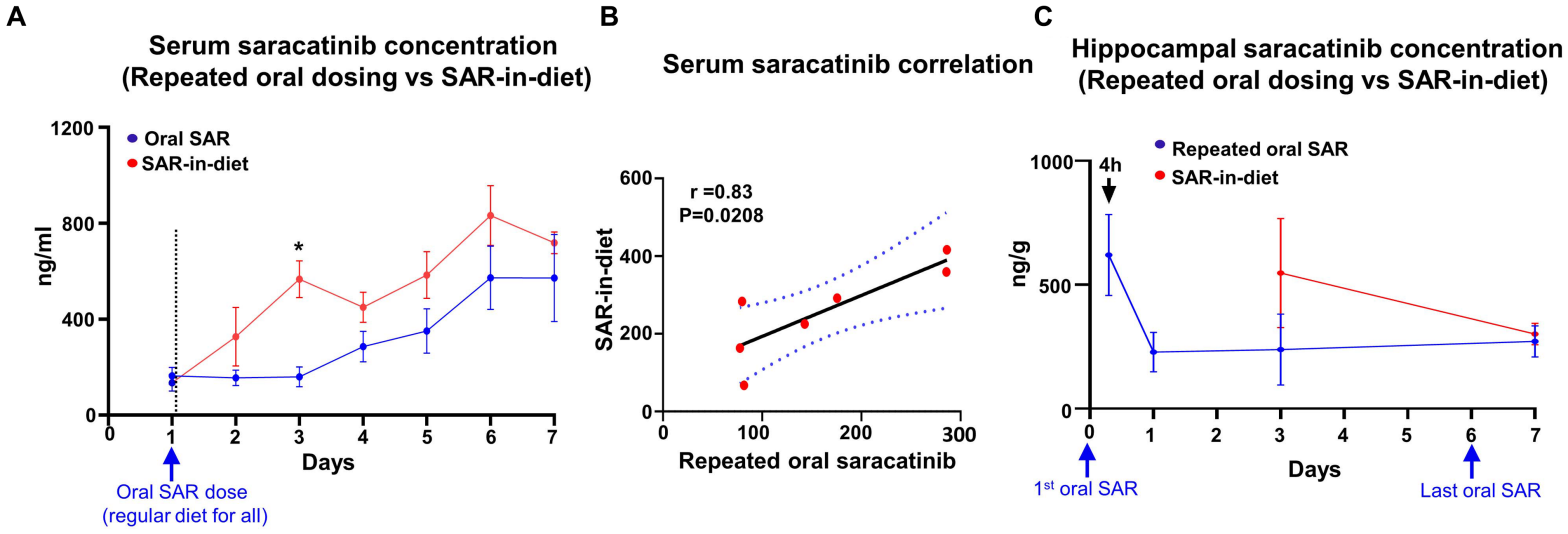
Comparison between mean (± SEM) serum concentrations of saracatinib after repeated oral administration (20 mg/kg) and in SAR-in-Diet (20 mg/kg) groups **(A)**. Correlation between the serum concentration of saracatinib between repeated oral administration (20 mg/kg) and in SAR-in-Diet (20 mg/kg) groups **(B)**. Hippocampal SAR concentrations **(C)**. Significant increase in the serum concentrations of SAR at only day 3 in SAR-in-Diet versus repeated oral dosing **(A)** *n* = 4, mixed-effects analysis (Šídák’s multiple comparisons test) *p < 0.05.

In the repeated daily dosing of saracatinib (20 mg/kg, oral gavage), blood sampling was done at 24 h intervals before the next oral dosing. The serum saracatinib concentrations were maintained at ~100 ng/mL for the first three days, while trough serum concentration of saracatinib steadily increased during 3–6 days to reach a plateau at day 7 (Figure 5A). In the SAR-in-Diet group, the serum concentration steadily increased during days 1–3, 5, and 6 with a reduction in saracatinib systemic levels at day 4 and day 7 (Figure 5A). However, there were no significant differences in the serum concentrations of saracatinib between the repeated daily oral dosing (group 2) and SAR-in-Diet (group 3) except on day 3 (Figure 5A), which could be due to reduction in food intake in the SAR-in-Diet group during the previous 24 h (Figure 2A). Overall, there was a strong and significant positive correlation in the serum concentrations of saracatinib between the repeated daily oral dosing and SAR-in-Diet groups (Figure 5B). Hippocampal saracatinib concentrations in the repeated oral dosing maintained nearly constant levels at days 3 and 7 (Figure 5C). There were no significant differences in the hippocampal saracatinib concentrations when compared between the repeated daily oral dosing versus the animals fed on SAR-in-Diet at 3 and 7 days (Figure 5C).

### 3.5. Serum concentrations of saracatinib when the animals were fed with different concentrations of SAR-in-Diet for long term

**Figure 6:**
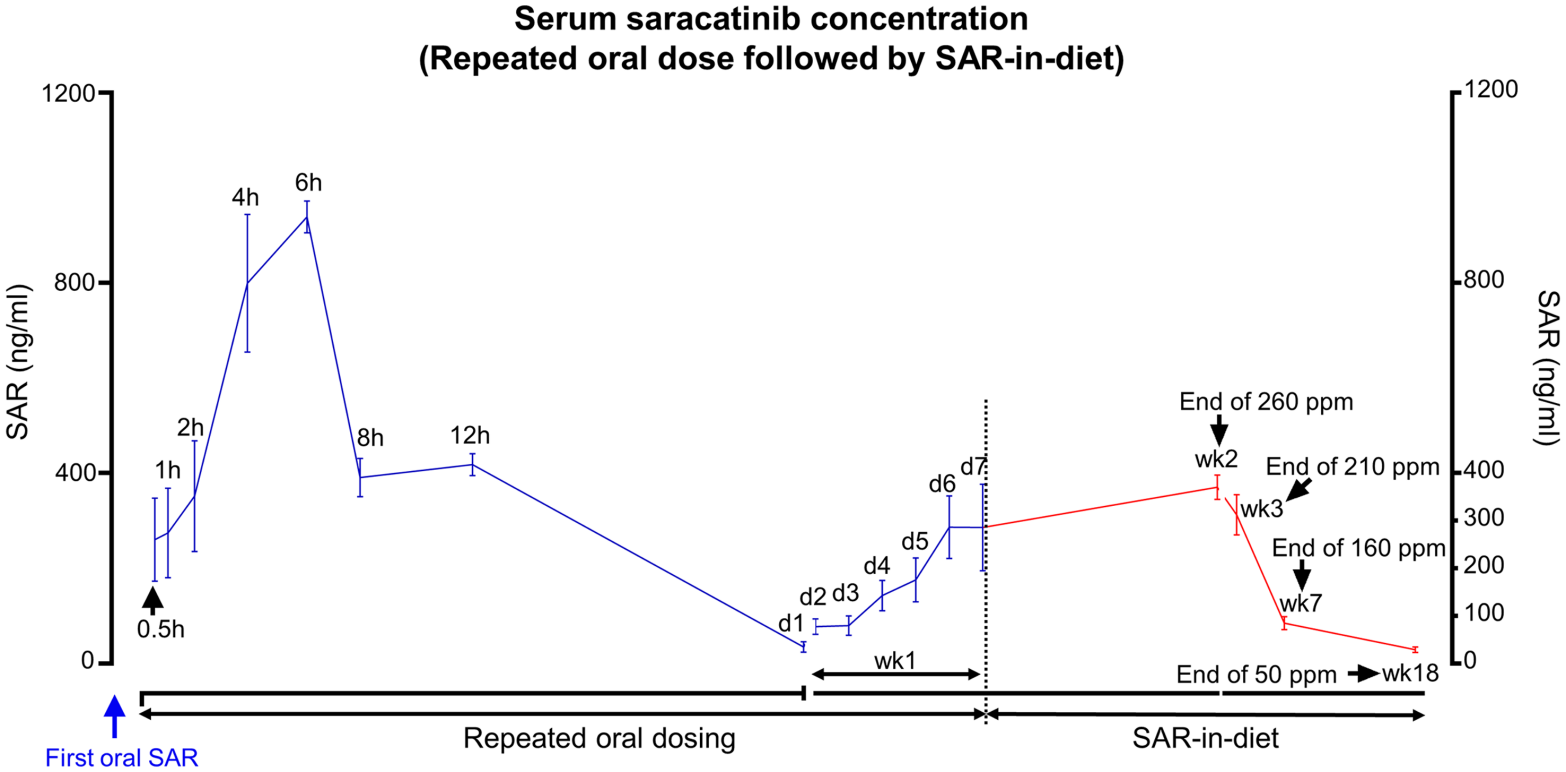
Serum SAR concentrations in animals that received repeated daily oral dosing of SAR (20 mg/kg) followed by varying concentrations of SAR-in-Diet for long term. *n* = 4–8.

SAR-in-Diet of different concentrations were prepared, and the drug concentrations were confirmed in the prepared diet by LC–MS/MS (Table 1). The animals first received daily oral dosing of saracatinib (20 mg/kg of rat) for a week and then fed with SAR-in-Diet of varying/tapering concentrations. The concentrations of saracatinib in the diet were 260, 210, 160, and 50 ppm and were given for 2, 1, 4, and 11 weeks, respectively (group 4). Blood samples were collected at the end of each period before the diet was changed. The experimental design for group 4 is illustrated in Figure 1D. The overview of the serum saracatinib concentrations is represented in Figure 6.

**Table 1:** SAR concentration in diet and SAR dose rate in long term study (18 weeks).

Expected SAR-in-Diet (ppm)	Actual SAR conc. (ppm)	Target dose rate of SAR (mg/kg)	Dose rate of SAR achieved (mg/kg)	No. of weeks on the diet
260	260.15	20	17.47	2
210	219.41	15	13.75	1
160	165.45	10	8.95	4
50	54.43	5	2.75	11

### 3.6. Correlation analysis of serum SAR levels and SAR-in-Diet consumption at different concentrations

**Figure 7:**
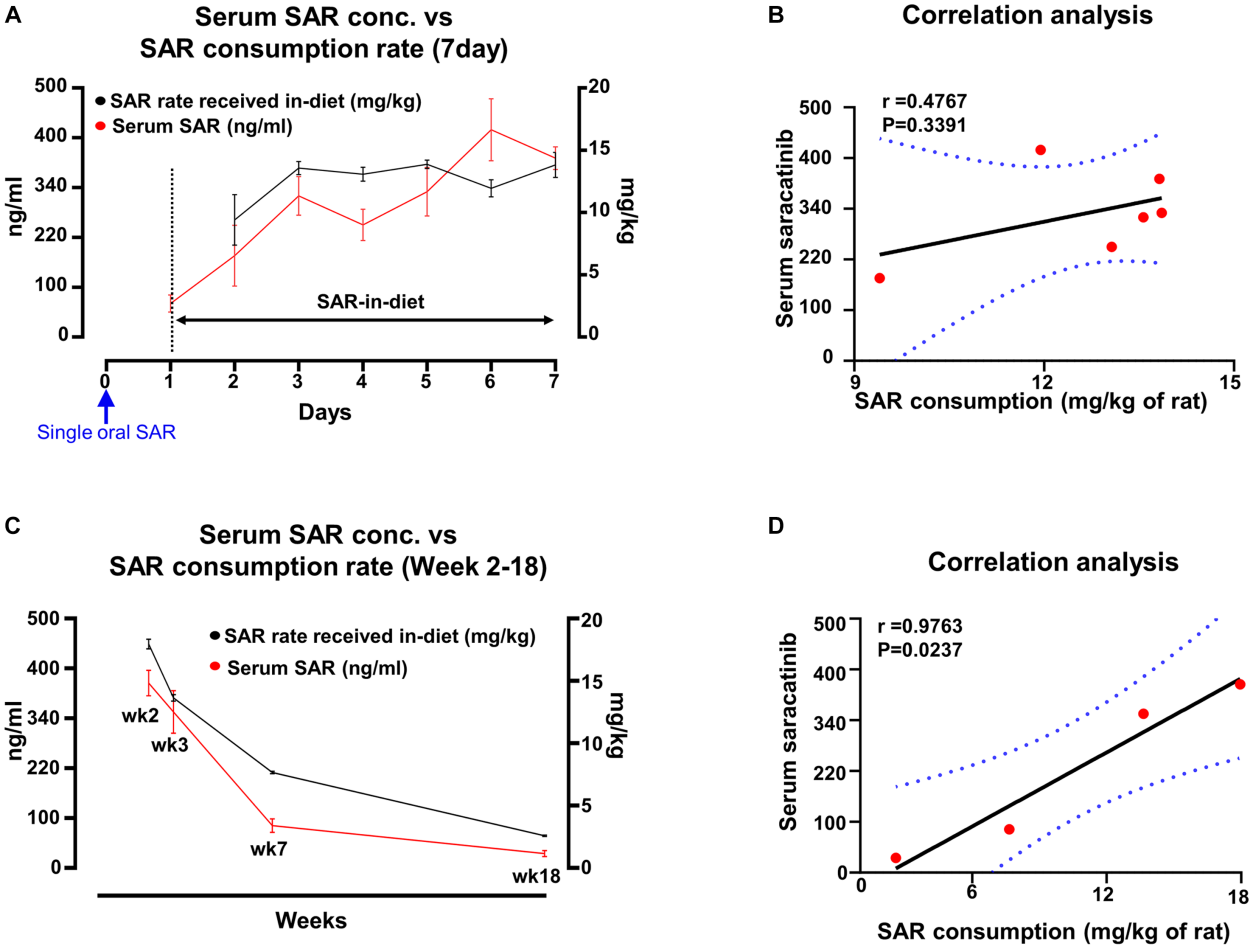
Comparison and correlation analysis of serum concentration of saracatinib and its consumption rate when the animals were fed with varying concentrations of SAR-in-Diet. *n* = 4–8. **(A,C)** mixed-effects analysis (Šídák’s multiple comparisons test); *n* = 4–8 **(B,D)** Pearson correlation analysis.

In group 3, animals were given saracatinib with an initial single oral dose followed by 6 days of SAR-in-Diet at 260 ppm. The serum concentration of saracatinib and the rate at which the drug was consumed (mg/kg of rat) via SAR-in-Diet is presented in Figure 7A. A positive correlation was observed between serum concentration and the SAR-in-Diet consumption rate (Figure 7B). In group 4, animals were given repeated oral dosing of saracatinib for 7 days followed by SAR-in-Diet at 260 ppm, 210 ppm, 160 ppm, and 50 ppm until the end of week 2, week 3, week 7 and week 18, respectively. The serum concentration of saracatinib and the rate at which the drug was received through SAR-in-Diet is presented in Figure 7C. Overall, a strong and positive significant correlation was observed between serum saracatinib concentrations and the drug consumption rate via the diet (Figure 7D).

## 4. Discussion

In this study, we demonstrated the feasibility of incorporating various concentrations of an orally active test drug saracatinib, for about 18 weeks in healthy adult male rats. We also compared the serum disposition kinetics, feed consumption, and weight gain between orally administered saracatinib and diet-incorporated saracatinib in a week-long study. Our descriptive PK analysis confirmed that saracatinib is well absorbed when administered orally, reaches a peak concentration at approximately 6 h, and is well-distributed to the brain. Clinical pharmacokinetics of saracatinib in healthy humans at the dose rate of 250 mg/day once a day daily for 10–14 days has shown excellent pharmacokinetic parameters with the T_1/2_ of 40 h and was well tolerated. However, the drug and its metabolites may accumulate in the tissues over time (20). Therefore, a tapering dosing regimen may mitigate such adverse effects in long-term studies. In contrast to human pharmacokinetics, our study in adult rats shows that the half-life of saracatinib was much lower in rats, perhaps due to a higher metabolic rate.

Exposure to DFP and soman can lead to status epilepticus resulting in spontaneous recurrent seizures (21–24). We have previously demonstrated the disease-modifying effects of saracatinib in the rat kainate and DFP models of epilepsy (3, 16). However, saracatinib efficacy seems to depend on the initial severity of the seizures and subsequent brain pathology (25). Therefore, optimizing the effective dose of saracatinib for chronic diseases such as epilepsy, AD, and certain types of cancers, and a long term treatment of saracatinib at decreasing concentrations may be required to minimize adverse effects. Src/Fyn kinases play a significant role in cell proliferation and migration, an active process in cancers and chronic neurodegenerative diseases (for example, gliosis). Therefore, inhibiting Src/Fyn kinase activity can be beneficial to control or modify the progression of the disease.

Saracatinib has been tested in several clinical trials for various types of cancers, AD and PD. In all these trials, saracatinib was administered as oral tablets or capsules for a very long time (13, 26, 27). Since saracatinib is an orally active drug, it has to pass through the hepatic circulation for its metabolism. A repeated oral gavage in experimental models is not feasible for long term. Furthermore, repeated handling of animals for oral gavage causes stress and confounds the experimental results. Therefore, for long term studies test drugs are normally added to the drinking water or the feed (28). In a mouse model of AD, saracatinib incorporated into the diet was fed for up to 9 months and demonstrated its disease-modifying effects (29). In the mouse AD study, they monitored the body weight to determine the food/drug intake. Our current study demonstrates the feasibility, stability, serum and brain pharmacokinetics of saracatinib when the animals were fed for the long term with the drug incorporated in the rat chow. We also compared the differences in serum and brain saracatinib concentrations between daily oral dosing versus SAR-in-Diet in a 7-day study. The brain concentrations were also decreased as the serum concentrations decreased but took a longer time to clear from the brain, which is an advantage from a therapeutic perspective.

The commonly used routes of drug delivery in laboratory animals require repeated handling and restraint that are aversive to animals. This can be a problem in studies that require chronic administration of drugs daily orally. Furthermore, the timing of administration of the test drug may impact the pharmacokinetics and the drug metabolism, which depends on animal’s metabolic state during the day or the night. Since rodents are nocturnal, they are metabolically active at night and eat more than during the day. This may be an advantage for rodents if a test drug mixed in the diet was fed, especially for epileptic conditions to control the incidence of nocturnal seizures.

Several studies from other labs have demonstrated the delivery of drugs such as antibiotics or disease-modifying agents in drinking water or by incorporating them into the diet (29, 30). However, the choice of such a delivery method depends on the stability of the test drug at room temperature and whether the test drug remains in solution without precipitation. Although saracatinib in solution is stable at room temperature for several weeks, it may require stirring to prevent precipitation, therefore, not suitable for delivering it in drinking water for long term treatment. Furthermore, when added to water, saracatinib solution alters its taste and impacts daily water consumption. Diet-incorporated saracatinib has been shown to be effective in a mouse model of AD (29). Repeated oral dosing or chronic diet incorporated delivery is required to achieve a steady state of saracatinib serum concentrations. Although we did not find significant differences between the two approaches, sustained and steady optimum serum concentrations of saracatinib are likely to be maintained through diet rather than acute dosing. Acute dosing rapidly increases serum concentrations of saracatinib within 6 h and decreases after that. Such rapid increases of test drug in serum may have off-target effects in contrast to a gradual buildup of serum concentrations when the test drugs were administered through the diet (31–33).

The choice of administration of test drugs in rodents depends on various factors such as rate of absorption, target retention/engagement, and the rate of metabolism, which is high in small rodents and impacts drug clearance (34, 35). Drugs administered in humans or other species with lower metabolic rates and various disease conditions require dosage adjusting to achieve optimum therapeutic levels in serum and the target organs (36). Our data from repeated daily oral dosing versus SAR-in-Diet suggest that a steady state of therapeutic concentration in serum can be achieved through diet incorporation.

The main objective of the translational studies in preclinical rodent models is to acquire adequate and robust scientific data for future drug development for clinical application. In humans, oral medication takes priority over parenteral routes of administration for obvious reasons. Therefore, characterizing the orally active test drugs in preclinical models has a translational potential. Saracatinib has been tested in various experimental models and its PK and toxicity at high doses are also known from clinical trials (8, 37). Our published work has demonstrated the disease-modifying effects of saracatinib in experimental models of epilepsy (3, 25). The long term treatment of saracatinib in epilepsy and AD models, and in various cancerous studies in clinical trials provide adequate evidence for the translational potential of saracatinib for future drug development. Furthermore, saracatinib reverses ATP-binding cassette (ABC) transporters B1 (ABCB1) mediated multidrug resistance (15). Interestingly, saracatinib only inhibits ABCB1 transport function, without altering ABCB1 expression or AKT phosphorylation. Therefore, incorporating orally active antiepileptic drugs and saracatinib in the diet could offer therapeutic benefits in epilepsy.

## 5. Conclusion

Repeated oral dosing of saracatinib and SAR-in-Diet produced comparable serum saracatinib concentrations with no significant differences between groups, except on day 3 wherein saracatinib serum concentrations were significantly higher in SAR-in-Diet group. Overall, saracatinib concentrations in the serum and the hippocampus reached therapeutic concentrations in rats fed a SAR-in-Diet, which has obvious potential benefits in the management of chronic diseases such as epilepsy. This approach can be extended to many other orally active therapeutic drugs that are intended for use long term.

## Data availability statement

The original contributions presented in the study are included in the article/Supplementary material, further inquiries can be directed to the corresponding author.

## Ethics statement

The animal study was approved by Iowa State University Institutional Animal Care and Use Committee (IACUC). The study was conducted in accordance with the local legislation and institutional requirements.

## Author contributions

TT: Conceptualization, Funding acquisition, Project administration, Resources, Supervision, Validation, Visualization, Writing – original draft, Writing – review & editing. SV: Conceptualization, Data curation, Formal analysis, Investigation, Methodology, Software, Validation, Visualization, Writing – original draft, Writing – review & editing. NM: Conceptualization, Data curation, Formal analysis, Investigation, Methodology, Software, Validation, Visualization, Writing – original draft, Writing – review & editing. SN: Methodology, Validation, Visualization, Writing – review & editing. JM: Methodology, Validation, Visualization, Writing – review & editing. LS: Data curation, Methodology, Writing – review & editing.
