# Supplementary material for: Exploring the benefits of in-diet versus repeated oral dosing of saracatinib (AZD0530) in chronic studies: insights into pharmacokinetics and animal welfare
Source: Front Vet Sci. 2023 Nov 9;10:1297221. doi: 10.3389/fvets.2023.1297221 (PMC10666625; doi:10.3389/fvets.2023.1297221)
Supplement: Supplementary file 1 [file Table_1.DOCX]

Table S1. Serum PK parameters for saracatinib following single dosing at 20mg/kg orally in rats (n=4)

| **Pharmacokinetic parameter** | **Median** | **Range** |
| --- | --- | --- |
| T_1/2_(h) | 4.12 | (2.95-5.34) |
| Kel (1/h) | 0.18 | (0.13-0.24) |
| Cmax (ng/mL) | 954.42 | (844.25-1001.68) |
| Tmax (h) | 6 | (6-6) |
| AUC _(0-tlast)_ (ng/mL*h) | 6992.15 | (6276.21- 7990.59) |
| AUC _(0-∞)_ (ng/mL*h) | 7061.97 | (6638.78- 8434.33) |
| V_d_/F (L/kg) | 15.59 | (12.06- 22.09) |
| CL/F (L/h/kg) | 2.83 | (2.37-3.01) |
